# Research Progress on Molecular Breeding and Application of *Clematis* Plants

**DOI:** 10.3390/plants14233575

**Published:** 2025-11-22

**Authors:** Jiehui He, Lin Lin, Yizeng Chen, Xule Zhang, Yaping Hu, Lei Feng, Xiaohua Ma, Jiayi Lin, Qingdi Hu, Jian Zheng

**Affiliations:** 1 College of Forestry and Biotechnology, Zhejiang A&F University, Hangzhou 311300, China; jiehuig@163.com; 2 Key Laboratory of Plant Innovation and Utilization, Engineering Research Center for Southeast Coastal Characteristic Plants of National Forestry and Grassland Administration, Zhejiang Institute of Subtropical Crops, Wenzhou 325005, China; linlin3100lin@163.com (L.L.); 13736366389@163.com (Y.C.); zhangxule2025@126.com (X.Z.); huyp@zaas.ac.cn (Y.H.); leifeng9305@gmail.com (L.F.); maxh@zaas.ac.cn (X.M.); 3 The First Clinical College of Chinese Medicine, Hunan University of Chinese Medicine, Changsha 410208, China; jiayilinwz@163.com

**Keywords:** *Clematis*, molecular breeding, ornamental character, stress resistance, development

## Abstract

*Clematis* L., a significant genus of climbing plants within the Ranunculaceae family, boasts widespread germplasm resources distributed across temperate to tropical regions globally, with Asia preserving particularly abundant native populations. This review systematically summarizes recent advances in *Clematis* research: in terms of physiological characteristics, the research focuses on the evolution of plant classification, chromosomal evolutionary features revealed by karyotype analysis, and studies on genetic diversity and phylogenetic relationships based on molecular markers; in breeding methods, it summarizes the two major technical systems of sexual and asexual reproduction; regarding ornamental traits, it emphasizes the molecular mechanisms of flower color and form development, and synthesizes breakthroughs in techniques for flowering period regulation and research on the biosynthesis pathways of floral scent metabolites; in the field of stress resistance mechanisms, it thoroughly examines physiological responses and molecular adaptation mechanisms under abiotic stresses such as UV radiation, drought, high temperature, and intense light, and outlines research progress on pathogen types of major diseases; in studies of medicinal value, it highlights the material basis and mechanisms of pharmacological activities including anti-inflammatory, analgesic, and antitumor effects. Through multidimensional comprehensive analysis, this review aims to elucidate the comprehensive development potential of *Clematis*, providing theoretical foundations and practical guidance for germplasm resource innovation, breeding of high-ornamental-value cultivars, and stress resistance applications.

## 1. Introduction

*Clematis* L., a significant perennial genus within the Ranunculaceae family, is widely distributed across temperate to tropical regions worldwide, with particularly abundant native germplasm resources found in Asia [1]. This genus exhibits considerable morphological diversity, encompassing various life forms such as woody or herbaceous vines, erect herbs, and shrubs. Renowned for its elegant flower forms and rich color variations, *Clematis* holds the esteemed title of “Queen of Climbers” and is extensively utilized in vertical greening, landscape gardening, and container cultivation for its high ornamental value. Furthermore, *Clematis* contains multiple bioactive compounds, including saponins, demonstrating pharmacological properties such as antitumor, anti-inflammatory, and antioxidant activities, thereby indicating considerable potential for medicinal plant development [2].

The genus *Clematis* comprises over 300 species worldwide [3]. Conventional *Clematis* breeding has primarily relied on hybridization and seedling selection, yet this process remains slow, inefficient, and constrained by certain interspecific reproductive barriers. In-depth studies on the taxonomy and karyotype analysis of *Clematis* provide a crucial foundation for overcoming these limitations. While classical morphological classification and modern molecular phylogenetic studies together establish a phylogenetic framework for species within the genus, karyotype analysis further reveals genetic structure, ploidy characteristics, and evolutionary relationships at the chromosomal level, offering essential cytological evidence for the effective utilization of wild germplasm in distant hybridization [4].

Moreover, despite the significant ornamental and medicinal value demonstrated by *Clematis*, its industrialization faces considerable challenges. On one hand, existing cultivated varieties still require substantial improvement in key ornamental traits—such as flower color, form, and blooming period—to meet increasingly diverse market demands. On the other hand, the relatively weak resistance of many horticultural varieties to abiotic stresses such as high temperature, drought, intense light, and diseases limits their widespread cultivation and landscape application in many regions worldwide [5]. Current breeding efforts in *Clematis* predominantly focus on phenotypic improvement of floral traits such as color and form, along with horticultural application development, while fundamental research on stress resistance traits and underlying molecular genetic mechanisms remains insufficient. The application of molecular breeding techniques can facilitate the development of specific cultivars, significantly enhancing the efficiency of targeted improvement for traits like heat-humidity tolerance and disease resistance, thereby increasing both economic returns and ornamental value. This review systematically summarizes recent advances in *Clematis* research, organizing progress across physiological characteristics, ornamental traits, stress tolerance, and applied development to provide a reference for further utilization and development of this genus.

## 2. Physiological Characteristics Research

### 2.1. Plant Classification

**Figure 1:**
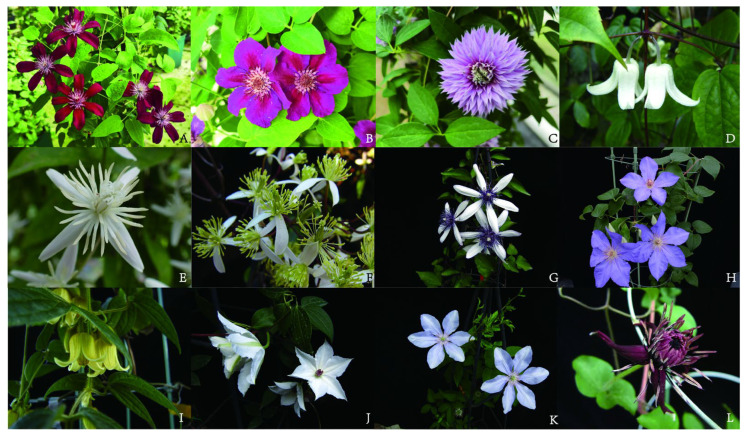
Illustrations of Selected *Clematis* Species and Cultivars. (**A**) *Clematis* ‘Stasik’, (**B**) *Clematis* ‘Ashva’, (**C**) *Clematis* JOSEPHINE ‘Evijohill’, (**D**) *Clematis henryi*, (**E**) *Clematis finetiana*, (**F**) *Clematis crassifolia*, (**G**) *Clematis akoensis*, (**H**) *Clematis lanuginosa*, (**I**) *Clematis leschenaultiana*, (**J**) *Clematis tientaiensis*, (**K**) *Clematis cadmia*, (**L**) *Clematis hancockiana*.

The genus *Clematis* L., a member of the Ranunculaceae family, exhibits remarkable species diversity and extensive morphological variation. Figure 1 shows illustrations of some *Clematis* species and cultivars., while the horticultural classification system is detailed in Supplementary Materials (Table S1). The taxonomic study of *Clematis* has undergone a prolonged evolution, progressing from classical morphological classification toward an integrated framework incorporating cytological, chemical, and molecular phylogenetic approaches [6]. The generic name *Clematis* was first established by Linnaeus in Species Plantarum. Early classification systems primarily relied on stable morphological traits—such as leaf type (simple/compound), inflorescence structure, morphology and texture of sepals, characteristics of stamens and carpels, and achene morphology with persistent style length—yet these criteria may present limitations in distinguishing certain species [7]. For instance, while *Clematis serratifolia* and *Clematis glauca* exhibit high morphological similarity and are difficult to differentiate based on external characteristics, embryological studies by Yang et al. revealed distinct differences in their early embryonic development, particularly in megaspore formation and nucellus type, suggesting a potential polyphyletic origin [8].

Recent phylogenetic analyses utilizing chloroplast genes (e.g., *rbcL*, *matK*, *atpB*) and nuclear genes (e.g., *ITS*, *PHYA*) have substantially revised and deepened our understanding of the systematic relationships within *Clematis*. In a study by S.X. Yan et al., two nuclear gene markers (*ITS* and *ETS*) and six chloroplast gene markers (*rps16*, *rpl16*, *accD*, *trnS-trnG*, *atpB-rbcL*, and *trnV-atpE*), combined with DNA barcoding techniques, were employed to assess the taxonomic status of *Clematis acerifolia* var. *elobata* and its relationship with other *Clematis* species. Results revealed an average genetic divergence of 0.78% between *Clemati acerifolia* var. *acerifolia* and *C. acerifolia* var. *elobata*, indicating clear differentiation and the formation of two distinct clades. Based on these findings, the study elevated *C. acerifolia* var. *elobata* to species level [9].Employing a non-targeted ultra-performance liquid chromatography-tandem mass spectrometry (UPLC-MS/MS)-based metabolomics approach, Jiedong Yan et al. conducted a comprehensive differentiation of *Clematis tangutica*, *Clematis intricata*, and Clematidis Radix et Rhizoma (CRR) collected from eight provinces in China. The results revealed that flavonol biosynthesis and flavonoid biosynthesis are closely associated with geographical origin, identifying flavonoid biosynthesis as a key biochemical marker for geographical adaptation in the genus *Clematis* [10].

The genus *Clematis* has been divided into several natural subgeneric groups based on integrated evidence from morphology, cytology, and molecular phylogenetics. This classification not only reflects its evolutionary history but also provides a theoretical foundation for germplasm utilization. Traditional morphological classification systems primarily relied on floral structure and growth habit for subgeneric delineation. As early as 1982, Carl S. Keener et al. classified the *Clematis* species of North America north of Mexico into four subgenera: *Clematis*, *Atragene* (L.) Torrey & Gray, *Viorna* A. Gray, and *Viticella* (Moench) Keener & Dennis, stat. nov [11]. Among these, only members of subgenus *Clematis* exhibit significant distribution extending into Latin America and the Caribbean, occurring in both lowland tropical habitats and the more temperate, high-altitude communities typical of Ranunculaceae [12]. Subsequent systematic studies have focused on the Viorna group within the genus. Through analyses incorporating morphological, anatomical, and palynological data, comparisons were made among 25 out of 76 species—16 from the Old World and 9 from North America—leading to the recognition of four subgenera: *Campanella* Tamura, *Tubulosa* T.Y.A. Yang nom. nov., *Viorna* A. Gray, and *Integrifolia* (R.O. Erickson) T.Y.A. Yang, stat. nov [13].

### 2.2. Karyotype Analysis

As early as 1939, researchers initiated karyotype studies in the genus *Clematis* [14]. Karyotypic variation within the genus can be classified into multiple types. In a study by Lu Sheng et al., chromosomal characteristics—including chromosome number, satellite number and position, karyotype formula, arm ratio, relative length, centromeric index, and karyotype asymmetry coefficient—were examined across 11 *Clematis* species. Results indicated that all analyzed species exhibited the same stable chromosome number (2n = 2x = 16) and a basic number of x = 8. Species such as *C. lanuginosa*, *Clematis otophora*, *Clematis lasiandra*, *Clematis shenlungchiaensis*, *Clematis florida* var. *plena*, *Clematis fusca* var. *violacea*, *Clematis crispa*, and *Clematis viticella* were classified under type 2A, representing a highly symmetrical and primitive karyotype. In contrast, *C. henryi*, *Clematis integrifolia*, and *Clematis japonica* were categorized as type 2B, reflecting a more evolutionarily derived condition compared to other groups [15].A study by Puneet Kumar et al. revealed that *Clematis flammula* has a chromosome number of 2n = 16, classifying it as a diploid. Its chromosomes exhibit a continuous decrease in length, ranging from 14.83 (±3.6) µm to 7.58 (±3.65) µm. The karyotype formula of this species is 2n = 2x = 16 = 10 m + 6 sm, which differs notably from that of the tetraploid species *Clematis orientalis* (2n = 4x = 32 = 18 m + 8 sm + 6 st). In terms of karyotype symmetry, the intrachromosomal asymmetry index (A1) of *C. flammula* is 0.39, significantly lower than that of *C. orientalis* (A1 = 0.96), indicating a more symmetrical karyotype in the former [16]. Jialun Ren et al. conducted karyotype analysis on root tip materials from 13 *Clematis* cultivars, including ‘Andromeda’, ‘The Vagabond’, ‘Hakaookan’, ‘Rhapsody’, and ‘Blue Light’. Results indicated that all examined cultivars were diploid, with a chromosome number of 2n = 2x = 16 and a base number of x = 8. Their karyotype formulas were highly similar, all corresponding to Stebbins’ type 2A. The karyotype asymmetry coefficients ranged narrowly from 61.78% to 64.78%, with cultivars such as ‘Alba Plena’, ‘Mme Julia Carrevon’, and ‘The Vagabond’ exhibiting relatively higher evolutionary advancement [17].

### 2.3. Genetic Diversity

Researchers have utilized high-throughput sequencing technologies to conduct sequencing and analysis of organelle genomes in multiple *Clematis* species. These studies have elucidated the overall architecture of the organelle genomes in certain *Clematis* taxa, revealing genetic variations and phylogenetic relationships among different species within the genus.

Jinping Qin et al. determined the complete chloroplast (cp) genome of *Clematis nannophylla* and subsequently reconstructed the phylogeny of the genus *Clematis*. The chloroplast genome has a total length of 159,801 bp and exhibits a typical quadripartite structure, comprising a large single-copy region, a small single-copy region, and a pair of inverted repeats (IRa and IRb). Phylogenetic analysis revealed that *C. nannophylla* is more closely related to *Clematis fruticosa* and *Clematis songorica* [18].Using the Illumina sequencing platform, Cui Yi et al. sequenced the complete chloroplast genome of *Clematis hexapetala*. The results demonstrate that the chloroplast genome is a circular DNA molecule with a total length of 159,538 bp, exhibiting a typical quadripartite structure composed of four regions: two IRregions (each 31,039 bp), a large single-copy region (LSC, 79,333 bp), and a small single-copy region (SSC, 18,127 bp). Phylogenetic analysis indicates a close relationship between *C. hexapetala* and *Clematis taeguensis* [19].Utilizing a combination of Illumina short-read and Nanopore long-read sequencing technologies, Dan Liu et al. sequenced and annotated the mitochondrial genome of *C. acerifolia*. This work provided the first complete characterization of the mitochondrial genome for this species and conducted a comparative phylogenetic analysis with its close relatives. The results revealed a total length of 698,247 bp for the *C. acerifolia* mitochondrial genome, with a primary multi-branched architecture comprising one linear and two circular molecules. Furthermore, the mitochondrial genome structure was found to be highly non-conserved, exhibiting extensive inter-organellar sequence transfers from the chloroplast, sequence duplications, and RNA editing events. Phylogenetic analysis demonstrated a close relationship between *C. acerifolia* and *Anemone maxima* [20].

In *Clematis* research, molecular marker techniques such as random amplifiedpolymorphim DNA (RAPD) and inter-simple sequence repeat (ISSR) have been widely employed. These methods analyze DNA sequence polymorphisms to reveal the genetic diversity within *Clematis* and the phylogenetic relationships among its cultivars. In 2013, researchers utilized the RAPD approach to authenticate a biologically derived traditional Chinese medicine prepared from plants of the *Clematis* genus [2]. In studies on genetic diversity, *Clematis* exhibits substantial genetic variation, which is manifested not only in DNA sequence polymorphisms but also in morphological characteristics, physiological and biochemical properties, as well as ecological adaptability. Zhigao Liu et al. developed simple sequence repeat (SSR) markers based on the transcriptome sequencing of *C. finetiana*. A phylogenetic tree constructed from the SSR data revealed that *Clematis brevicaudata* and *Clematis apiifolia* belong to the same clade, while *C. uncinata* is closely related to *C. finetiana* [21]. Xin Wang et al. employed ISSR technology to analyze the genetic diversity of 17 *Clematis* varieties, comprising five wild species and 12 Texas cultivars. Their analysis classified the five wild *Clematis* species into two distinct clades: one clade corresponds to the Section *Clematis* group, including *Clematis pinnata*, *Clematis tubulosa*, and *C. brevicaudata*, while the other clade belongs to the subgenus Urophylla, containing *C. fusca* and *Clematis reticulata* [22]. Liuwangsou determined the complete chloroplast genome sequence of *Clematis tomentella* and conducted an analysis of its interspecific relationships. The results indicate that the chloroplast genome of *C. tomentella* has a total length of 159,816 bp, consisting of twoIR regions, each 31,045 bp in length, aLSC region of 79,535 bp, and a SSC region of 18,191 bp. Further analysis identified significant sequence variation in the *psbE*-*petL*, *trnG*_UCC-*atpA*, *ndhF*-*rpl32*, and rps8-infA regions, which can serve as potential DNA barcodes for species identification. Moreover, a maximum likelihood phylogenetic tree revealed that *C. tomentella* is most closely related to *C. fruticosa* [23]. Zhengnan Zhao et al. assessed the genetic diversity of *C.* *acerifolia* using SSR markers. The results revealed substantial genetic differentiation between *Clematis* *acerifolia* var. *elobata* and *C. acerifolia*, with intra-population genetic variation being the primary source of population variation in *C. acerifolia* [24].

To date, beyond traditional molecular marker techniques, no large-scale studies have comprehensively explored the genetic background of *Clematis*. Genotyping-by-sequencing (GBS) enables de novo genotyping of breeding populations and facilitates the construction of accurate genomic selection models, even for large, complex, and polyploid genomes. Yaping Hu et al. employed GBS to investigate the genetic diversity and structure of 103 *Clematis* accessions (35 wild species and the remainder horticultural cultivars). STRUCTURE analysis classified all accessions into two subpopulations (C1 and C2). The results demonstrated higher genetic diversity among the horticultural cultivars than within the wild populations. Extensive artificial hybridization has not only facilitated the selection of new cultivars but also substantially enriched their genetic background. Furthermore, the genetic differentiation between horticultural cultivars and wild species was significantly greater than that observed between subpopulations C1 and C2. Regarding effective population size, horticultural cultivars have exhibited a gradual increasing trend since approximately 800 years ago, whereas the wild populations have experienced a continuous, slow decline [25].

Furthermore, while traditional perspectives posit speciation as a slow and gradual process primarily driven by the accumulation of genetic mutations, a growing body of empirical evidence indicates the occurrence of rapid speciation in plants, wherein hybridization—particularly homoploid hybridization—plays a significant role in driving rapid adaptation and speciation. By integrating phylogenomic, morphological, and ecological data, Jian He et al. demonstrated that *Clematis* sect. *Fruticella* and the related sect. Meclatis originated from ancient homoploid hybridization events. The genomic composition of the hybrid descendants combined key adaptive traits from their parental lineages, facilitating the colonization of novel ecological niches and sustaining subsequent diversification. This finding indicates that ancient hybridization acted as a combinatorial mechanism that played a pivotal role in the adaptive evolution of this group [26].

## 3. Ornamental

Recent years have witnessed notable progress in functional gene and transcription factor research in *Clematis* (Table 1), providing a theoretical foundation for molecular breeding in this genus. Current genetic improvement efforts for ornamental *Clematis* primarily focus on two key objectives: enhancing horticultural value by improving ornamental traits such as flower color, form, and abundance; and employing molecular-assisted breeding techniques to bolster plant resistance to both biotic stresses (e.g., diseases and pests) and abiotic stresses (e.g., extreme climates), while simultaneously expanding their ecological adaptability.

### 3.1. Flower Color

The formation of core ornamental traits in *Clematis*—such as flower color, form, flowering period, and plant architecture—is governed by multi-layered regulatory networks. RNA-seq technology has been widely applied to analyze gene expression across different growth stages, tissue types, and environmental conditions in *Clematis*. Liu Zhigao et al. investigated anthocyanin accumulation and the expression patterns of related genes during various floral developmental stages in two *Clematis* species, *C. hancockiana* and *Clematis courtoisii*. A total of 11 anthocyanins were identified in the red sepals of *C. hancockiana*, with cyanidin-3-O-glucuronide-malonyl-arabinoside being the predominant component. Concurrently, the expression of several structural genes involved in anthocyanin biosynthesis—including *ClF3’H*, *ClF3’5’H*, *ClDFR*, and *ClANS*—was significantly upregulated. In contrast, no anthocyanins were detected in the white sepals of *C. courtoisii*, and the expression levels of the aforementioned structural genes were extremely low. Furthermore, correlation analysis revealed that the regulatory genes *ClMYB22*, *ClMYB12*, *ClMYB28*, and *ClMYB4* showed significant positive correlations with multiple anthocyanin structural genes, suggesting their potential role in positively regulating anthocyanin accumulation [27].

Renjuan Qian et al. demonstrated that cyanidin-3-O-galactoside and cyanidin-3-O-sophoroside promote anthocyanin accumulation in *Clematis titaiensis*, with *WDR2* playing a significant regulatory role in anthocyanin biosynthesis [28]. Mingxia Yuan et al. conducted quantitative analyses of six core anthocyanin components in double-flowered *Clematis* cultivars representing four major color groups, revealing significant specificity in anthocyanin metabolic profiles among the different colored varieties: delphinidin was most abundant in blue cultivars, while cyanidin showed the highest concentration in red cultivars [42]. Further research indicates that secondary metabolites in *Clematis* associated with flower color formation—including total phenols, tannins, proanthocyanidins, flavonoids, and anthocyanins—all exhibit substantial antioxidant activity and free radical scavenging capacity. These compounds contribute to delaying aging, protecting cardiovascular and cerebrovascular health, mitigating inflammatory responses, and demonstrate a certain degree of anti-cancer effects [43]. Xiaozhu Guo et al. investigated multiple compounds, differentially expressed genes (DEGs), and flavonoid biosynthesis-related pathways in *C. tangutica* from two distinct ecological environments. The results indicated that variations in flavonoid content are the primary cause of differences in floral coloration between the two habitats. Acacetin and genkwanin were identified as the two flavonoid compounds exhibiting the most significant differences in floral composition between the two environments. Transcriptome analysis revealed that *BZ1-1* and *FG3-1* are key genes for delphinidin-3-O-rutinoside in anthocyanin biosynthesis, while *HCT-5* and *FG3-3* are critical genes for acacetin and naringin in the flavonoid and flavone/flavonol biosynthesis pathways, respectively [44].

### 3.2. Pattern

In studies on floral organ regulation, Xuerong Chen et al. demonstrated that ABCE model genes play a crucial role in the morphogenesis of *Clematis* flowers, with low expression of *ClAG2* potentially being a key factor promoting the formation of double flowers in *Clematis* [30]. Ying Wang et al. found that the seasonal variation in flower forms of *C.* ‘Vyvyan Pennell’ is associated with genes such as *VRN1*, *SVP*, and *Hd3a*. Furthermore, gibberellic acid (GA_3_), salicylic acid (SA), and trans-zeatin riboside (tZR) may influence the formation of double perianths in spring, whereas gibberellin GA_7_ and ABAmay affect the development of single flowers in autumn [31].Furthermore, altitude also influences floral traits. Xiang Zhao et al. investigated floral characteristics and sex allocation patterns in *C. tangutica* along an elevational gradient in the eastern Qinghai Plateau. The results demonstrated that with increasing elevation, sepal length, width, thickness, and weight, stamen and pistil length, androecium mass, the sex allocation ratio (A/G, representing the ratio of male to female investment), and seed set all increased. In contrast, the number of stamens and pistils, gynoecium mass, and fruit set decreased. However, the underlying molecular mechanisms remain unclear [45].

### 3.3. Flowering Time Regulation

Current approaches to flowering regulation in *Clematis* primarily rely on hormonal control, though functional characterization of key regulatory genes and elucidation of their signaling pathways remain limited. Some researchers suggest that plant growth regulators can promote *Clematis* development, while temperature and light manipulation may accelerate growth and shorten the production cycle. In a study on *C.*’H.F. Young’, Uttara *Clematis* Samarakoon et al. reported that increasing cold treatment duration from 0 to 9 weeks while extending photoperiod from 9 to 16 h resulted in elevated numbers of both buds and flowers, accompanied by reduced time to flowering [46]. Mingjian Chen et al. demonstrated that the TM3 and AGL6 subfamilies within the MADS gene family play significant roles in floral organ development and flowering time regulation in *C. finetiana* [32].

### 3.4. The Fragrance of Flowers

In floral fragrance research, Jiang Yifan et al. revealed that terpene synthase (TPS) influences terpenoid biosynthesis, while O-methyltransferase (OMT) and SABATH methyltransferase are potentially involved in the synthesis of benzenoids, fatty acid derivatives, and other volatile compounds [47]. Furthermore, their transcriptomic sequencing of *C. florida* ‘Kaiser’ flowers identified *CfTPS1* and *CfTPS2* among the detected TPS genes, which catalyze the conversion of geranyl diphosphate to linalool—the principal aromatic component in ‘Kaiser’ [33].

## 4. Stress Resistance

The genus *Clematis* frequently encounters various abiotic stresses (e.g., drought, salinity, high temperature, low temperature) and biotic stresses (e.g., pathogenic diseases) across its natural distribution and cultivation. Its stress resistance mechanisms directly influence the survival and distribution of germplasm resources, as well as the breadth of horticultural applications, making this field a current research focus.

### 4.1. Ultraviolet Stress

*Clematis* species are widely distributed across diverse ecological environments, and their capacity to adapt to environmental stresses is crucial for survival. Coumarins, belonging to the phenylpropanoid class of compounds, can enhance plant stress tolerance by bolstering antioxidant defense systems. Previous studies have demonstrated that coumarin content increases in *Clematis terniflora* under ultraviolet stress [48].Minglei Tao et al. identified the key coumarin biosynthetic enzyme *CtF6’H* in *C. terniflora* and confirmed its function and enzymatic properties through heterologous expression in *Escherichia coli* [34]. Furthermore, their proteomic analysis of the UV stress response mechanism in *Clematis* demonstrated that ultraviolet radiation B (UV-B) exposure damages the photosynthetic system and reduces total chlorophyll content in *C. terniflora* leaves. UV-B treatment also increased the protein abundance of sucrose synthase (SUS) and sucrose phosphate synthase (SPS), while ultraviolet D (UVD) treatment elevated both SUS and sucrose phosphatase (SPP) levels, suggesting that plants may reprogram carbohydrate metabolism to maintain cellular homeostasis under ultraviolet stress. Additionally, ultraviolet stress activated the mitogen-activated protein kinase (MAPK) signaling pathway and enhanced the abundance of phosphoproteins associated with osmotic stress response [49].

### 4.2. Drought Stress

Drought tolerance varies among different *Clematis* cultivars. Using five wild *Clematis* species (*C. tangutica*, *C. glauca*, *C. intricata*, *C. nannophylla*, and *C. fruticosa*) as experimental materials, Zhuzhu et al. simulated varying intensities of drought stress through polyethylene glycol (PEG) treatment and observed seed germination behavior and physiological-biochemical responses in seedlings. The results demonstrated that *C. tangutica* exhibited the strongest drought tolerance. Under drought stress, malondialdehyde (MDA) content, soluble sugar content, and soluble protein content increased with stress intensity, while chlorophylls (Chls) and carotenoid (Car) concentrations initially increased then decreased. These response strategies may represent protective mechanisms for plant adaptation to arid conditions [50].

Salt stress and drought stress often interact in natural environments, with appropriate salt pretreatment potentially enhancing drought resistance by regulating plant osmotic adjustment mechanisms and antioxidant defense systems. Within this context, Wenli Zhang et al. systematically measured the physiological responses to drought in *C. fruticosa* seedlings under low salt (150 mM NaCl) and salt-free conditions. The results revealed that as drought stress duration extended, both leaf relative water content and photosynthetic pigment content in *C. fruticosa* showed declining trends, while MDA content exhibited an initial increase followed by a decrease. The low salt pretreatment significantly improved drought tolerance in *Clematis* seedlings by promoting the accumulation of osmoregulatory substances and maintaining antioxidant enzyme activity [29].

### 4.3. Heat Stress

High-temperature stress disrupts plant cell membrane integrity, leading to increased electrical conductivity and elevated MDA content [51]. In response to elevated temperatures, *C. lanuginosa* cells activate a series of molecular response mechanisms. These include the upregulation of heat shock protein (HSP) encoding genes, enhanced involvement of the chloroplast ribosomal pathway, and modulation of the regulatory pathway from heat shock transcription factors to heat shock proteins [5].Qingdi Hu et al. investigated the potential regulatory mechanisms underlying heat stress response in *C. crassifolia* and *C. cadmia*. Their findings indicate that heat stress induces leaf discoloration while reducing net photosynthetic rate, stomatal conductance, and the activities of catalase and superoxide dismutase. Following heat stress, the expression of HSP and HSF genes, including *c194329_g3* and *c194434_g1*, was significantly upregulated in *C. cadmia*. Concurrently, transcript levels of four heat stress-responsive genes involved in photosystem and chlorophyll biosynthesis—*c200811_g3*, *c188817_g1*, *c187075_g1*, and *c194962_g2*—were elevated in *C. crassifolia* [35].

Renjuan Qian et al. conducted a comparative analysis of differentially expressed genes in response to heat stress between *C. crassifolia* and *C. lanuginosa*. The results demonstrated that serine/glycine/threonine metabolism, glyoxylate metabolism, and thiamine metabolism represent crucial pathways for heat stress response in *C. lanuginosa*, while flavonoid metabolism, phenylalanine metabolism, and arginine/proline metabolism constitute key pathways in *C. crassifolia* [52]. Changhua Jiang et al. revealed that heat shock proteins and the antioxidant system play significant roles in the thermotolerance of *C. florida*. Fifteen DEGs showed increased expression at both mRNA and protein levels in the heat-tolerant group, including two heat shock proteins (Hsp18 and Hsp70). Furthermore, genes such as *GLUST*, *GO1*, *RPE3*, *P5PI3*, *RbcS*, and *POD4* were implicated in carbohydrate metabolism and antioxidant pathways [36]. Rui Wang et al. demonstrated that the heat shock transcription factor *CvHSF30-2* enhances thermotolerance in *Clematis vitalba* by upregulating HSP expression following heat stress [37]. Through integrated transcriptomic comparison between heat-sensitive and heat-tolerant *Clematis* varieties, phylogenetic identification, and functional validation, hao Zhang et al. revealed the critical role of *CvHSFA2-2*: its heterologous expression in yeast enhanced heat resistance, while silencing its homologous gene *NbHSFA2* in tobacco compromised thermotolerance, thereby providing key insights into the heat response mechanisms of *Clematis* [38].

### 4.4. Waterlogging Stress

*Clematis* plants generally exhibit poor waterlogging tolerance, and direct research in this area remains limited, though waterlogging constitutes a key stress factor affecting their cultivation survival. Using *C. tientaiensis*, *C. lanuginosa*, ‘Sen-No-Kaze’, and ‘Viva Polonia’ as plant materials, Chen Kai et al. investigated their physiological responses and gene expression profiles under waterlogging stress, and evaluated the effect of exogenous melatonin application on waterlogging tolerance. The results indicated that ‘Sen-No-Kaze’ and *C. lanuginosa* displayed relatively weaker waterlogging tolerance. Under waterlogging stress, relative electrical conductivity and H_2_O_2_ content significantly increased in *Clematis*; photosynthetic parameters and chlorophyll content markedly decreased; photosynthesis was inhibited; and soluble protein and soluble sugar contents were reduced. Exogenous melatonin application effectively enhanced waterlogging tolerance, and transcriptome sequencing identified nine transcription factors—including *LBD4*, *MYB4*, *bHLH36*, and *DOF36*—with potential roles in improving waterlogging tolerance in *Clematis* [39].

### 4.5. Light Intensity Stress

Xiaohua Ma et al. found that under high irradiance conditions, *Clematis* growth was inhibited while chlorophyll levels increased, along with elevated activities of catalase, peroxidase, and superoxide dismutase. Expression of *c144262_g2*, *c133872_g1*, and *c142530_g1* decreased in irradiance-tolerant *C. cadmia*, whereas their expression increased in irradiance-sensitive *C. crassifolia*, indicating these genes are associated with light environment response in *Clematis* [40]. Furthermore, by investigating physiological responses and gene expression in *C. tientaiensis* under four light treatments (1800 ± 30/0 µmol m^−2^ s^−1^; 1500 ± 30/0 µmol m^−2^ s^−1^; 1200 ± 30/0 µmol m^−2^ s^−1^; 900 ± 30/0 µmol m^−2^ s^−1^), they analyzed its potential adaptation mechanisms to light intensity. The results showed that strong light caused leaf chlorosis and yellowing, reduced net photosynthetic rate (Pn), stomatal conductance (Gs), Rubisco activase enzyme (RAC) and Rubisco enzyme activities, while increasing the content of eight amino acids. As light intensity decreased, the expression levels of *psbA*, *psbB*, *psbC*, and *Psb(OEC)* genes were down-regulated [41].

### 4.6. Biological Stress

*Clematis* leaf spot and blight is a common disease, where infected plants initially develop irregular brown to black leaf spots that progressively expand into large necrotic lesions, ultimately leading to blight. Using leaf, stem, and root tissues from diseased plants as experimental materials, M. Špetík et al. cultured pathogenic fungal mycelia on potato dextrose agar (PDA) medium and obtained purified strains through single-spore isolation. Among the isolated strains, 21 exhibited morphological characteristics similar to fungi of the genus *Calophoma*. For further identification, the researchers amplified genomic DNA fragments including the internal transcribed spacer (ITS), large ribosomal subunit (LSU), β-tubulin gene (*tub2*), and the gene encoding the second largest subunit of RNA polymerase II (*rpb2*). The resulting sequences were compared with corresponding reference sequences—ITS (NR_135964), LSU (FJ515632), *tub2* (FJ427100), and *rpb2* (KT389588)—from the *Calophoma clematidina* type strain CBS 108.79. The results demonstrated complete sequence identity between all amplified fragments and the reference sequences, confirming *C. clematidina* as the causal agent of *Clematis* leaf spot and blight [53].Rujun Zhou et al. Identified *Erysiphe aquilegiae* from diseased tissue through morphological characterization and rDNA ITS sequence analysis of powdery mildew-infected *Clematis manshurica*, marking the first formal report of *E. aquilegiae*-caused powdery mildew on *Clematis* in China [54]. In 2022, the first occurrence of rust disease on large-flowered *Clematis* was reported in Korea. Based on morphological characteristics, sequence alignment data, and pathogenicity test results—where the ITS sequence showed only a single nucleotide difference from *Coleosporium clematidis* (KX386005) and the LSU sequence also exhibited a single nucleotide variation compared to *C. clematidis* sequences (KX386039, KX386040, and KX386042)—the pathogen was confirmed to be *C. clematidis* [55].

## 5. Breeding and Propagation Techniques of New Cultivars

### 5.1. New Cultivar Breeding

As an important ornamental genus, systematic breeding of new *Clematis* cultivars has been carried out in numerous countries, with the United Kingdom, Poland, the United States, Japan, and China representing the primary research nations in this field. While conventional cross-breeding remains the predominant approach, modern biotechnology primarily serves a supplementary role. In recent work, Chinese researchers developed a distinct red-purple cultivar, ‘Violet Lipstick’, through stem cutting propagation of *Clematis texensis* ‘Cherry Lip’ seedlings. This cultivar produces solitary, campanulate, and monoecious flowers 2–2.5 cm in diameter, borne singly in leaf axils or at shoot tips, with a slightly nodding to pendulous habit. Its four sepals are elliptic, thick, and fleshy in texture, displaying a deep red-purple coloration [56]. Molecular markers offer a means for efficient selection of superior cultivars during breeding. In a study by Linfang Li et al., 127 *Clematis* germplasm accessions from nine countries were analyzed using 44 EST-SSR and 22 sequence-related amplified polymorphism (SRAP) primers for genome-wide association studies. The analysis detected 133 polymorphic EST-SSR loci and 81 polymorphic SRAP loci. Association mapping via general linear and mixed linear models identified 6 and 7 markers, respectively, significantly linked to heat tolerance, providing crucial theoretical and technical support for marker-assisted selection in breeding heat-tolerant *Clematis* cultivars and accelerating the development of new cultivars adapted to high-temperature environments [57].

Current research progress indicates that although molecular biological methods have found certain application in fundamental studies of *Clematis*, the development of commercial cultivars through transgenic or gene-editing technologies continues to encounter numerous challenges. Key technical limitations include: the genetic transformation system for *Clematis* still requires optimization, with stable regeneration protocols and efficient transformation methods yet to be fully established; furthermore, the structural characteristics of its genome may introduce additional complexities for genetic engineering operations.

### 5.2. Sexual Propagation Techniques

Sexual reproduction serves as a significant propagation method in *Clematis*, enabling the production of vigorous seedlings with well-developed root systems and enhanced stress tolerance. These progenies not only exhibit considerable genetic diversity but also retain desirable maternal traits to a certain degree. However, seeds of some *Clematis* species display varying degrees of dormancy during sexual propagation. Breaking such dormancy can improve reproductive efficiency to some extent, with common methods including stratification, exogenous hormone application, and organic reagent treatments. Previous studies have demonstrated that exogenous GA_3_ treatment promotes seed germination [58].Furthermore, the thick seed coat of *Clematis* seeds often leads to physical dormancy. Moharam Ashrafzadeh et al. demonstrated that mechanical scarification with sandpaper effectively breaks dormancy in *Clematis ispahanica*; their study further indicated that sowing depth significantly influences germination rates, with optimal results achieved at a density of 1.12 kg per hectare (approximately 30 seeds/m^2^) and a depth of 1.5 cm [59]. Subsequent research by Keliang Zhang et al. revealed that external environmental conditions, particularly temperature and light, affect seed germination in different *Clematis* varieties during sexual reproduction. Their findings showed that *C. hexapetala* seeds initiated germination after 6 weeks of cold stratification at 4 °C, with germination rates progressively increasing with extended stratification duration. Moreover, higher germination rates were observed under alternating 12 h light/dark cycles compared to complete darkness [60].

### 5.3. Asexual Propagation Techniques

(1) Cutting propagation. Cutting propagation serves as a key method for cultivar multiplication in the genus *Clematis*. During the process, the selection of both the rooting medium and the cuttings themselves is critical. In a study by Silja Kreen et al., microshoots and softwood stem cuttings from five *Clematis* varieties (Clematis section Atragene) were rooted using different substrates (perlite, peat-perlite, and sand-perlite). The results consistently demonstrated that microshoots exhibited superior rooting efficiency and speed compared to stem cuttings, regardless of substrate type. Furthermore, the root dry mass of microshoots exceeded that of stem cuttings by more than twofold. For stem cuttings, the highest rooting percentage and speed were observed in pure perlite (71%), while peat-perlite yielded the lowest (36%). The maximum number of primary roots and root dry mass for stem cuttings were recorded in both sand-perlite and pure perlite substrates [61].

The rooting success of *Clematis* cuttings is jointly influenced by genetic characteristics, endogenous hormone levels in the cuttings, the physiological condition of the stock plants, and environmental conditions during propagation. Research by Uttara C. Samarakoon et al. demonstrated that stock plants maintained at 27 °C produced subsequently rooted cuttings with increased quantity and dry weight compared to those grown at 21 °C. Furthermore, propagation using shoots derived from underground buds resulted in approximately 50% greater cutting yield and higher fresh and dry weights of rooted cuttings relative to those obtained from axillary buds. Additionally, management practices including pruning stock plants to substrate level while maintaining long-day photoperiods and warm temperatures (27 °C) promoted the development of young, non-lignified shoots suitable for cutting propagation [62].In a study by N.A. Prokhorova et al. assessing the propagation efficiency of *Clematis* green cuttings in Western Siberia, the cultivars ‘Ville de Lyon’ (93%), ‘Madame Baron Veillard’ (82%), and ‘Purpurea Plena Elegans’ (80%) demonstrated the highest rooting capacity [63].

(2) Tissue Culture. Tissue culture represents a significant approach for the asexual propagation of *Clematis* species. Compared to methods such as cutting and layering, this technique is not constrained by environmental conditions and offers advantages including rapid propagation rates and high multiplication coefficients. Various explant types can be utilized in *Clematis* tissue culture, with stem segments, seeds, leaves, and roots being commonly used. The choice of explant varies among different *Clematis* species. Furthermore, the selection of culture medium is crucial for successful propagation. A study by Danuta Kulpa et al. demonstrated that MS medium supplemented with 1 mg∙dm^−3^ 6-benzylaminopurine (BAP) achieved effective microshoot induction in *Clematis* ‘Warszawska Nike’, with a success rate of 80%. Notably, higher BAP concentrations in the medium promoted substantial callus formation at the explant base. For in vitro root induction, the highest acclimatization survival rate (75%) was observed when using MS medium containing 0.5 mg∙dm^−3^ indole-3-acetic acid (IAA) [64]. Research conducted by Irina Mitrofanova et al. evaluated the influence of BAP and thidiazuron (TDZ) on morphogenetic capacity across 13 clematis cultivars. Their findings revealed that media containing either BAP or TDZ supported the highest regeneration frequency of morphologically n ormal shoots from callus, with optimal cytokinin concentrations identified as 4.40 μM BAP and 6.0 μM TDZ for micropropagation. During somatic embryogenesis, illumination at 37.5 μmol m^−2^ s^−1^ was found to promote increased somatic embryo production [65].

## 6. Research on Medicinal Effects

### 6.1. Anti-Inflammatory and Analgesic Effects

*Clematis* species, recognized in traditional Chinese medicine, demonstrate significant therapeutic potential for inflammatory and pain conditions. Their anti-inflammatory and analgesic properties have been extensively validated in both traditional clinical practice and modern pharmacological research. The pharmacological activity primarily derives from triterpenoid saponins and flavonoids as key bioactive constituents. These bioactive constituents exert therapeutic effects through multi-target and multi-mechanism synergistic actions, such as downregulating inflammatory factors and modulating signaling pathways. This multi-faceted activity reveals substantial potential for treating rheumatoid diseases, traumatic injuries, and infectious inflammations, thus establishing *Clematis* as a valuable natural medicinal resource worthy of further development [66].

Contemporary research has transitioned from traditional applications in treating superficial inflammations and pain to elucidating molecular mechanisms involving cytokine regulation and signaling pathways. Current studies have demonstrated that *Clematis* triterpenoid saponins (CTSs) can significantly alleviate arthritis symptoms in collagen-induced arthritis (CIA) rats, modulate intestinal microbiota imbalances, and downregulate short-chain fatty acid (SCFA) concentrations, suggesting their potential to ameliorate rheumatoid arthritis through the restoration of gut dysbiosis [67]. Ting Lei et al. revealed that the anti-inflammatory mechanism of *C. florida* likely involves the modulation of the NF-κB and MAPK signaling pathways, leading to reduced expression of IL-6, COX-2, TNF-α, and IL-1β [68]. These findings are consistent with the results reported by Shuzhong Xu et al. [69]. Nana Yang et al. found that the *C. florida* Active Fraction (CFAF) significantly alleviates arthritis symptoms in adjuvant-induced arthritis (AA) rats, including reduced paw swelling, lowered arthritis index, and improved histopathological conditions, while also effectively decreasing serum levels of IL-1β, TNF-α, and IL-6. The underlying anti-inflammatory mechanism involves triterpenoid saponin components in CFAF inhibiting the production of inflammatory mediators in lipopolysaccharide (LPS)-induced macrophages by blocking the JAK/STAT signaling pathway [70].

While existing studies have established a foundation for understanding their pharmacological effects, current research has predominantly focused on crude extracts. Future investigations should prioritize the isolation and purification of individual active compounds, elucidate their structure-activity relationships with precision, and identify specific molecular targets. This systematic approach will provide a solid scientific basis for developing novel anti-inflammatory and analgesic agents from these natural resources.

### 6.2. Antitumor Effect

The antitumor potential of *Clematis* represents a promising direction in modern pharmacological research, with its active constituents functioning through multiple synergistic pathways. Saponins, particularly triterpenoid saponins, demonstrate the ability to inhibit tumor cell proliferation and induce apoptosis. For instance, a novel bidesmosidic triterpenoid saponin isolated from the roots of *Clematis* uncinata exhibited inhibitory effects on human cervical cancer Caski cells [71]. Furthermore, a new flavonol compound,kaempferol3-O-[(6-O-caffeoyl)-glucosyl(1→2)]-(6-O-caffeoyl)glucoside-7-O-rhamnoside (6), along with six known flavonol molecules, were isolated from an ethanolic extract of *C. flammula* leaves. In cytotoxicity assays, this extract displayed significant antitumor activity against human hepatocellular carcinoma HepG2, ovarian cancer A2780, and OVCAR3 cell lines [72]. In their investigation of potential anti-tumor mechanisms, Zhu Furong et al. examined the anti-proliferative effects of saponins derived from *Plena Clematis* (PC) cultivated in China’s Fujian Province on four human tumor cell lines. The prepared saponins showed notable cytotoxic activity against human EC9706, KB, BGC-823, and HepG-2 cell lines [73].

At the molecular level, the antitumor effects of *Clematis* fundamentally involve precise modulation of multiple tumor-associated signaling pathways. Contemporary molecular pharmacology research continues to elucidate deeper mechanisms of action. A study by Manal I. Alruwad et al. demonstrated that the ethanol extract of Clematis cirrhosa (CEE) induced G2/M phase cell cycle arrest (19.63%) in HT-29 colorectal cancer cells, significantly triggered apoptosis (41.99%), and suppressed cell migration/wound healing by 28.15%. These effects were accompanied by upregulated expression of pro-apoptotic markers BAX (6.03-fold) and caspase-3 (6.59-fold), alongside reduced expression of anti-apoptotic BCL-2 [74].Research by Cheng Lin et al. revealed that *Clematis* hederagenin saponin (CHS) significantly downregulates the expression levels of mitochondrial apoptotic protease-activating factor-1 (Apaf-1) and cytochrome c proteins in breast cancer cells, while concurrently enhancing the activities of caspase-3 and caspase-9, ultimately inducing apoptosis through modulation of the mitochondrial apoptotic pathway [75].

### 6.3. Antioxidant Effect

The antioxidant capacity of *Clematis* species constitutes a fundamental basis for their pharmacological activities. This property not only correlates with their traditional medicinal value but also provides scientific rationale for developing modern functional therapeutics. Oxidative stress, characterized by an imbalance between reactive oxygen species (ROS) and the endogenous antioxidant defense system, is closely associated with various pathological processes including aging, neurodegenerative disorders, cardiovascular diseases, and cancer. Bioactive constituents abundant in *Clematis*, such as flavonoids, phenolic acids, and triterpenoid saponins, demonstrate remarkable antioxidant activity through multiple pathways including direct free radical scavenging and activation of endogenous antioxidant defense systems, thereby forming the material basis for their anti-inflammatory, antitumor, and anti-aging effects. Ismat Nawaz et al. evaluated the antimicrobial and antioxidant properties of leaf extracts from *Clematis* *montana* and *Clematis grata*. Their research revealed that methanol extracts from both species exhibited inhibitory activity against pathogenic bacteria (particularly *Pseudomonas aeruginosa* and *E. coli*), with no significant variations observed between different extraction methods (maceration/Soxhlet extraction). Further analysis indicated that *C. grata* methanol extracts possessed the highest total phenolic content and 1,1-Diphenyl-2-picrylhydrazyl (DPPH) radical scavenging capacity, demonstrating superior antioxidant activity [76]. In vitro experiments by M. Mostafa confirmed that leaves of *Clematis brachiata.* serve as a valuable source of natural antioxidants [77]. The investigation led by Ehsan Karimi and colleagues demonstrated that at 300 μg/mL, the ethyl acetate extract of *C. orientalis* and the chloroform extract of *C. ispahanica* exhibited the strongest DPPH-scavenging and ferric reducing/antioxidant potential (FRAP) reducing activities among all tested fractions [78].

### 6.4. Other Functions

Beyond their anti-inflammatory, antioxidant, and antitumor properties, *Clematis* species demonstrate additional pharmacological activities including antimicrobial, antiviral, and diuretic effects. These findings substantially expand their medicinal value and provide scientific support for potential applications in broader therapeutic areas [79, 80]. Research by M.E. Álvarez et al. indicated that macerates from the roots and aerial parts of *Clematis* *montevidensis* exhibit diuretic activity, primarily attributed to its characteristic constituent oleanolic acid [81]. Pengcheng Tu et al. revealed that CTSs promote cartilage repair through mechanisms involving inhibition of vascular endothelial cell activation and macrophage inflammation, coupled with enhanced chondrogenesis in bone marrow stromal cells [82]. Sankar Narayan Sinha’s investigation demonstrated that ethanol extracts from *Clematis gouriana* leaves significantly inhibit *Micrococcus luteus* and *Shigella dysenteriae* [83]. Previous studies have identified a novel mannose-binding lectin (designated CML) isolated from stems of *C. montana* with dual antiviral and antitumor activities. Regarding antitumor effects, CML markedly suppressed proliferation in L929, HeLa, MCF7, and HepG2 cells through caspase-dependent apoptosis induction. Crucially, this activity was strictly dependent on its mannose-binding capacity: complete blockage of CML’s carbohydrate-binding sites by sugars drastically reduced cytotoxicity and abolished apoptosis in L929 cells [84].

## 7. Conclusions and Future Perspectives

As an important plant resource with both ornamental and medicinal value, *Clematis* species have gained significant insights into their phylogeny and trait analysis through molecular markers and transcriptome studies. However, molecular breeding research is undergoing a critical transition from traditional breeding to precision breeding, while still facing numerous challenges: the lack of whole-genome information limits the study of evolutionary history, trait formation mechanisms, and breeding potential; low genetic transformation efficiency hampers the widespread application of gene editing technologies; and the polygenic regulatory mechanisms underlying key traits remain incompletely understood.

Future developments in *Clematis* molecular breeding should focus on the following directions: accelerating whole-genome sequencing and functional gene research, integrating existing molecular markers related to flower color, flower morphology, and stress resistance, and establishing a theoretical foundation for molecular design breeding; optimizing genetic transformation and gene editing technology systems to build efficient and safe genetic modification platforms; developing a “genotype-active component-efficacy” association database for medicinal value exploration, and using synthetic biology techniques to construct engineered systems for the efficient production of specialized active compounds such as triterpenoid saponins and flavonoids; and finally, achieving the transition from “experience-based breeding” to “precision design breeding” through the integration of multi-omics data and AI predictive models. This developmental pathway will not only provide technical support for the efficient utilization of *Clematis* germplasm resources but also offer a referential research model for the synergistic development of ornamental and medicinal plants.

## Figures and Tables

**Table 1:** Statistics of functional genes research of *Clematis*.

Function	Gene Name	Literature Source
Color control	*ClF3’H3*, *ClF3’5’H*, *ClDFR*, *ClANS*, *WDR2*, *BZ1-1*, *FG3-1*, *HCT-5*, *FG3-3*	Liuzhigao et al., 2025 [27]; Qian et al., 2023 [28]; Zhang et al., 2020 [29]
Flower pattern regulation	*ClAG2*, *VRN1*, *SVP*, *Hd3a*	Chen et al., 2025 [30]; Wang et al., 2024 [31]
Regulation of flowering	The TM3 subfamily and AGL6 subfamily in the MADS gene family	Chen et al., 2024 [32]
Floral fragrance regulation	*CfTPS1*, *CfTPS2*	Jiang et al., 2020 [33]
Ultraviolet (UV) stress	*CtF6′H*	Tao et al., 2023 [34]
High temperature stress	*HSP*, *HSF*, *GLUST*, *GO1*, *RPE3*, *P5PI3*, *RbcS*, *POD4*, *CvHSF30-2*, *CvHSFA2-2*, *NbHSFA2*	Hu et al., 2021 [35]; Jiang et al., 2020 [36]; Wang et al., 2021 [37]; Zhang et al., 2021 [38]
Waterlogging stress	*LBD4*, *MYB4*, *bHLH36*, *DOF36*, *WRKY4*, *MOF1*, *DOF47*, *REV1*, *ABR1*	Chen et al., 2024 [39]
Light intensity stress	Genes *c144262_g2* encoding the core receptor protein of the phytochrome system II, and genes *c133872_g1* and *c131300_g2* of the abscisic acid(ABA) receptor family *psbA*, *psbB*, *psbC*, *psb*(*OEC*)	Ma et al., 2019 [40] Ma et al., 2023 [41]

## Data Availability

No new data were created or analyzed in this study. Data sharing is not applicable to this article.

## Supplementary Materials

The following supporting information can be downloaded at: https://www.mdpi.com/article/10.3390/plants14233575/s1, Table S1: Horticultural Classification System of *Clematis*.
