# Supplementary material for: Research Progress on Molecular Breeding and Application of Clematis Plants
Source: Plants (Basel). 2025 Nov 22;14(23):3575. doi: 10.3390/plants14233575 (PMC12693783; doi:10.3390/plants14233575)
Supplement: Supplementary file 1 [file plants-14-03575-s001.zip › plants-3944862-supplementary.pdf]

Supplement Table S1. Horticultural Classification System of *Clematis*.

Category			
Small-flowered Division	Flat Small-flowered Subdivision	Armandii Group	Abundant 4–10 cm flowers with 4–6 white or pink sepals.
		Atragene Group	Bell-shaped flowers 2–12 cm with slender, drooping sepals in white, pale yellow or pink.
		Forsteri Group	Dioecious; flowers flat or bell-shaped, 2–9 cm across, with 4–8 white or yellowish-green sepals.
		Heracleifolia Group	Single, star-shaped tubular flowers with 4–6 white, creamy-yellow or reddish-purple sepals.
		Montana Group	Flowering in spring, it produces single, semi-double, or double blooms 3–14 cm across with 4–6 flat sepals in white, pink, deep magenta, or pale yellow.
		Vitalba Group	It typically produces single blooms about 5-6cm across, with 4-6 flat sepals in white or pale yellow.
		Viticella Group	Flowers single to double/bell-shaped, 2.5–12 cm, 4–6 sepals in white, pink, red, purple, violet or blue, often striped.
	Bell Small-flowered Subdivision	Integrifolia Group	Single, often bell-shaped flowers with 4–7 white, pink or reddish-purple sepals.
		Cirrhosa Group	Single pendent bell/bowl flowers with 4–5 creamy sepals, occasionally striped red or purple.
		Tangutica Group	Single bell-shaped or flat, often pendent flowers 2.5–9 cm with 4–6 white, yellow, orange or lilac sepals.
		Texensis Group	Single tulip- or bell-shaped flowers 4–10 cm with 4–6 thick pink, red or reddish-purple sepals.
		Viorna Group	Urn- or bell-shaped flowers 1.5–5 cm with 4 thick pink, pale purple or purple sepals.
Large-flowered Division		Early Large-flowered Group	Single to double flowers 7–25 cm with 4–9 white, cream, green, yellow, pink or red sepals.
		Late Large-flowered Group	Single, sometimes semi-double or double flowers 5–20 cm with 4–8 white, pink, pinkish-purple or red sepals.
